# Thunderstorm‐related asthma can occur in New Zealand

**DOI:** 10.1002/rcr2.655

**Published:** 2020-08-31

**Authors:** Ayan Sabih, Claire Russell, Catherina L. Chang

**Affiliations:** ^1^ Department of Respiratory Medicine Waikato Hospital Hamilton New Zealand; ^2^ Anglesea Accident and Medical Centre Hamilton New Zealand

**Keywords:** Asthma, thunderstorm

## Abstract

Thunderstorm asthma is induced by specific weather conditions causing breakdown and widespread distribution of allergens. Thunderstorm asthma had previously been considered unlikely to occur in New Zealand (NZ), given its local weather patterns. Storm events on 2 December 2017 led to increased asthma presentations at Waikato Hospital in Hamilton. Analyses of patient presentations led us to conclude that these presentations were similar to international descriptions of thunderstorm asthma. This is the first time such presentations have been reported in NZ. Documenting these events accurately is important as this is the first step to making a plan that would enable paramedics and emergency facilities across NZ to respond to any larger scale thunderstorm asthma events in the future.

## Graphical abstract

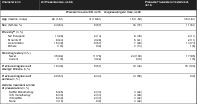

## Introduction

Thunderstorm asthma is an asthma‐related symptom brought about by the combination of specific weather conditions and the presence of high allergen concentrations. It is often but not necessarily associated with thunderstorms and can result in a large acute increase in the number of asthma‐related presentations. Importantly, people with little history of asthma can be affected, leading to delays in their presentation, diagnosis, and treatment.

The postulated mechanism of thunderstorm asthma involves pollen grains being lifted into clouds by storm updrafts typically in the late spring to early summer when the pollen count is high. Within the cloud, pollen grains are fragmented in the presence of water vapour into small particles. These small particles are then dispersed by the storm down‐drafts. Intact pollen grains, typically 12–60 μm, are trapped in the nasopharynx and upper airways and may cause allergic rhinitis. The fragmented pollen particles are much smaller (<10 μm) and can pass into the small airways where they can induce allergen‐mediated bronchoconstriction. Electrostatic deposition of pollen fragments may also occur in the small airways due to charge imbued on the fragments by the weather conditions [1]. Consequently, patients presenting with thunderstorm asthma may give a history of allergic rhinitis but often have no prior asthma diagnosis or previously well‐controlled asthma [2].

## Case Series

A thunderstorm on 2 December 2017 was followed by an increased number of acute asthma exacerbation presentations at after‐hours healthcare facilities in the Waikato region of New Zealand (NZ). Waikato Hospital is the only secondary care provider for Hamilton and surrounding areas. The following data were collected by reviewing all the medical records of presentations to emergency services from 0000 h 2 December 2017 till 0000 h 4 December 2017.

Fourteen adults (eight females, mean age: 31, range: 19–61) presented with asthma symptoms to the emergency department (ED) of Waikato Hospital during this period and gave clear histories of symptom onset during or following the storm. Ten of these 14 patients did not require inpatient treatment. Fifty percent of these ED‐discharged patients (5/10) only had a prior history of allergic rhinitis, and developed asthma symptoms for the first time after the storm while five patients had a previous diagnosis of asthma. All 10 patients rapidly improved following bronchodilator and oral steroid treatment and were discharged from the ED. Four patients required inpatient management due to the severity of their symptoms, and were admitted overnight under the respiratory medicine service. Three of these four patients gave a history of allergic rhinitis but no prior diagnosis of asthma. The fourth patient was 34 weeks pregnant, had a prior history of hay fever, and had been recently diagnosed with asthma during the pregnancy. All four patients gave clear histories of outdoor exposure at the time of the storm. They were formally diagnosed as cases of thunderstorm asthma by the attending respiratory physician. All of these patients rapidly improved following standard treatment for asthma exacerbation including systemic corticosteroids, inhaled corticosteroids, and short‐acting inhaled beta‐agonists.

For comparison, over a similar period in the preceding year (0000 h 2 December 2016 till 0000 h 4 December 2016), only five patients presented to the Waikato ED with asthma‐related symptoms and only one patient required hospital admission.

Anglesea Urgent Care is the only facility other than Waikato Hospital in the Hamilton region that provides 24‐h care. The authors were given access to patient records from 0000 h 2 December 2017 till 0000 h 4 December 2017 (day of the thunderstorm and 24 h afterwards), as well as the same period in December 2016 for a baseline comparison.

Anglesea Urgent Care managed 24 cases of asthma exacerbation presentations over the 48‐h period from 0000 h 2 December 2017 till 0000 h 4 December 2017. Of the 24 cases, 10 (42%) had an established diagnosis of allergic rhinitis, but no asthma prior to this event. They all reported typical asthma exacerbation symptoms for the first time in their lives after the storm. The remaining 14 patients had an established asthma diagnosis previously, and presented with an exacerbation during this time frame. In comparison, the same time period in 2016 saw half the number of patients (*n* = 11) presenting at the clinic. A summary of patient details is presented in Table 1.

**Table 1:** Patient characteristics of all acute asthma presentations.

Characteristic	All presentations, *n*=38			Probable thunderstorm asthma^*^, *n*=18
		Waikato Hospital ED, *n*=14	Anglesea Urgent Care, *n*=24	
Age (median, range)	26 (1–61)	31 (19–61)	15 (1–50)	35 (1–61)
Sex (female, %)	24 (63)	8 (57)	16 (37)	11 (61)
Ethnicity^†^ (*n*,%)				
NZ European	11(29)	2 (14)	9 (38)	2 (11)
Maori and PI	9 (24)	4 (29)	5 (21)	2 (11)
Asian/Indian	15 (39)	8 (57)	7 (29)	13 (73)
Others	3 (8)	0 (0)	3 (13)	1 (6)
Smoking history (*n*,%)				
Never	35 (92)	11 (79)	24 (100)	17 (95)
Current	3 (8)	3 (22)	0 (0)	1 (5)
Previous diagnosis of allergic rhinitis (*n*,%)	18 (48)	8 (57)	10 (42)	18 (100)
Previous diagnosis of asthma (*n*, %)	20 (53)	6 (43)	14 (58)	0 (0)
Asthma treatment at the time of presentation (*n*, %)				
SABA monotherapy	5 (25)	2 (33)	3 (22)	
ICS monotherapy	6 (30)	2 (33)	4 (29)	
ICS/LABA	6 (30)	2 (33)	3 (22)	
None	3 (15)	0 (0)	3 (22)	

* Patients were deemed to have probable thunderstorm asthma if they have all of the following: no previous diagnosis of asthma, a diagnosis of allergic rhinitis, and presented for the first time with asthma symptoms within 24 h of weather event.

† Ethnicity data were extracted from the medical records using the National Health Index entry. Other ethnicities include African, Middle Eastern, and mixed.

ED, emergency department; ICS, inhaled corticosteroid; LABA, long‐acting beta‐2 agonist; NZ, New Zealand; PI, Pacific Island; SABA, short‐acting beta‐2 agonist.

Anecdotally, emergency medical attendants and local general practitioners (GPs) interviewed by the authors also reported that there were increased asthma presentations in the four to five days following the thunderstorm event. These were often patients who did not have an existing asthma diagnosis and presentations were delayed because they did not know how to interpret the symptoms. A formal review of the local emergency respondent call‐outs was not conducted.

## Discussion

Although thunderstorm asthma is a recognized entity, cases are likely under‐reported because small outbreaks may go unnoticed [1]. Cases tend to only make headlines when sufficient cases requiring emergency care overwhelm the capacity of local services. A recent epidemic occurred in Melbourne in November 2016 when 8500 patients sought emergency care following a thunderstorm during peak grass pollen season, and at least 10 asthma‐related deaths and many intensive care unit (ICU) admissions were linked to this outbreak [3]. However, not all storms lead to asthma exacerbations. Weather conditions currently known to be associated with the development thunderstorm asthma include strong updraft convections, lower temperatures, storms with rainfall and moderate wind speeds, background grass pollen count of greater than 50 grains/m^3^/day, and storms with higher lightning events.

Following the Melbourne outbreak, there was local speculation on whether similar cases can occur in NZ. Interestingly, a national newspaper took the view that “while possible, such an event was unlikely in NZ” [4]. On the other hand, the greater weather extremes that occur as a consequence of climate change may lead to increased weather‐related asthma exacerbations. These exacerbations may go unrecognized unless they occur in large numbers. Local meteorological records showed wind gusts of up to 37 km/h combined with 770 lightning strikes during the storm that precipitated the asthma exacerbations. Unfortunately, there were no detailed aeroallergen data available. To our knowledge, this is the first reported case series of thunderstorm asthma in NZ.

We also observed a high proportion of people of Asian or Indian descent in our cohort. This is in keeping with previous reports of the Melbourne event in 2017 [3]. However, limitations to this observation must be acknowledged as the ethnicity data were retrospectively extracted from the National Health Index database rather than taken prospectively from the affected patients.

A significant proportion of people affected by thunderstorm asthma do not have a prior history of asthma. They are at higher risks of poor outcome during an asthma exacerbation as they do not have prior education to recognize symptoms and seek appropriate medical attention. They also do not have ready access to reliever bronchodilator inhalers. These patients are likely to present late and have more severe exacerbations on presentation. Management of these patients is also likely to be challenging as they do not give a history of established asthma with increased potential for misdiagnosis and thus ineffective treatment. Many patients presenting simultaneously may also overwhelm local healthcare services. Following the Melbourne outbreak, calls have been made to establish a public warning system and increased community resources [5]; however, current weather models lack sufficient specificity for the establishment of such a system.

In conclusion, the number of asthma presentations to emergency and urgent care increased more than twofold compared to historical volumes over the 24 h following a thunderstorm on 2 December 2017 in Hamilton, NZ. We describe a case series of 18 patients with classical features of thunderstorm asthma with no previous asthma history, prominent symptoms of allergic rhinitis, and exposure to outdoor conditions. This figure is likely an underestimate as many affected do not develop severe enough symptoms requiring immediate treatment. To our knowledge, this is the first description of thunderstorm asthma in NZ. Recognition and appreciation of this phenomenon will better enable local healthcare services to prepare, anticipate, and respond to these outbreaks.

### Disclosure Statement

Appropriate written informed consent was obtained for publication of this case report and accompanying images.
